# The Role of Robot-Assisted, Imaging-Guided Surgery in Prostate Cancer Patients

**DOI:** 10.3390/cancers17091401

**Published:** 2025-04-23

**Authors:** Leonardo Quarta, Donato Cannoletta, Francesco Pellegrino, Francesco Barletta, Simone Scuderi, Elio Mazzone, Armando Stabile, Francesco Montorsi, Giorgio Gandaglia, Alberto Briganti

**Affiliations:** 1 Unit of Urology, Division of Oncology, Gianfranco Soldera Prostate Cancer Lab, URI, IRCCS San Raffaele Scientific Institute, 20132 Milan, Italy; 2 Vita-Salute San Raffaele University, 20132 Milan, Italy

**Keywords:** prostate cancer, prostate-specific membrane antigen, radioguided surgery, augmented reality, sentinel node biopsy, near-infrared PSMA tracers

## Abstract

Emerging imaging-guided technologies, such as prostate-specific membrane antigen radioguided surgery (PSMA-RGS) and augmented reality (AR), could enhance the precision and efficacy of robot-assisted prostate cancer (PCa) surgical approaches, maximizing the surgeons’ ability to remove all cancer sites and thus patients’ outcomes. Sentinel node biopsy (SNB) represents an imaging-guided technique that could enhance nodal staging accuracy by leveraging lymphatic mapping with tracers. PSMA-RGS uses radiolabeled tracers with the aim to improve intraoperative lymph node metastases (LNMs) detection. Several studies demonstrated its feasibility and safety, with promising accuracy in nodal staging during robot-assisted radical prostatectomy (RARP) and in recurrence setting during salvage lymph node dissection (sLND) in patients who experience biochemical recurrence (BCR) after primary treatment and have positive PSMA positron emission tomography (PET). Near-infrared PSMA tracers, such as OTL78 and IS-002, have shown potential in intraoperative fluorescence-guided surgery, improving positive surgical margins (PSMs) and LNMs identification. Finally, augmented reality (AR), which integrates preoperative imaging (e.g., multiparametric magnetic resonance imaging [mpMRI] of the prostate and computed tomography [CT]) onto the surgical field, can provide a real-time visualization of anatomical structures through the creation of three-dimensional (3D) models. These technologies may assist surgeons during intraoperative procedures, thus optimizing the balance between oncological control and functional outcomes. However, challenges remain in standardizing these tools and assessing their impact on long-term PCa control. Overall, these advancements represent a paradigm shift toward personalized and precise surgical approaches, emphasizing the integration of innovative strategies to improve outcomes of PCa patients.

## Simple Summary

New imaging-guided technologies might improve outcomes of robot-assisted surgery for prostate cancer (PCa) patients. Specifically, sentinel node biopsy (SNB) can improve nodal staging by mapping lymphatic drainage, while prostate-specific membrane antigen radioguided surgery (PSMA-RGS) can enhance lymph node metastases (LNMs) identification through radiolabeled tracers during robot-assisted radical prostatectomy (RARP) and in the context of salvage lymph node dissection (sLND) for biochemical recurrence (BCR) after primary treatment. Moreover, near-infrared PSMA tracers like OTL78 and IS-002 can provide better intraoperative visualization, reducing positive surgical margins (PSMs) rates and improving LNMs detection. Additionally, augmented reality (AR), which integrates preoperative imaging (multiparametric magnetic resonance imaging [mpMRI] of the prostate, computed tomography [CT]) into three-dimensional (3D) models, can offer real-time guidance for decreasing the PSMs rate and maximizing functional outcomes during nerve-sparing surgery (NSS). Overall, these technologies could lead to more precise and personalized treatments, aiming to improve disease control and functional outcomes.

## 1. Introduction

Robot-assisted radical prostatectomy (RARP) represents one of the most commonly adopted treatment options for the management of prostate cancer (PCa) patients with localized or locally advanced disease [1]. Achieving a negative surgical margins status and removing all sites of lymph node metastases (LNMs) during an extended pelvic lymph node dissection (ePLND) are key to maximizing local disease control and, thus, to providing long-term cancer control. The presence of lymph node invasion (LNI) and positive surgical margins (PSMs) is indeed associated with an increased risk of biochemical recurrence (BCR) [2,3]. Despite technological advancements, a fundamental limitation of RARP lies in the inability to intraoperatively visualize the precise location of intraprostatic tumor and LNI in real time. In this context, preoperative imaging could be used for surgical planning. Multiparametric magnetic resonance imaging (mpMRI) of the prostate depicts an excellent specificity (91%) but a low sensitivity (51%) for detecting extra-capsular extension (ECE) at final pathology [4]. Additionally, information derived from prostate mpMRI increases the accuracy of the prediction of adverse pathological findings after radical prostatectomy (RP), namely ECE and seminal vesicle invasion (SVI) [5], which is crucial when a nerve-sparing surgery (NSS) is planned [6]. Accurate nodal staging is fundamental to identifying pN1 patients who may benefit from surgical treatment [7]. Prostate-specific membrane antigen positron emission tomography (PSMA PET) demonstrates higher accuracy compared to conventional imaging modality (CIM) [8]. However, its performance characteristics remain suboptimal, with sensitivity not exceeding 60% [9], primarily due to its limited reliability in detecting small LNMs (micro-metastases < 3 mm) [10,11]. Recently, intraoperative imaging-guided surgery showed promising results in assisting surgeons intraoperatively during robot-assisted surgery to ensure better oncological and functional outcomes [12]. Of particular interest, PSMA-radioguided surgery (PSMA-RGS) and augmented reality (AR) have been developed as advance intraoperative imaging-guidance tools. PSMA-RGS employs radiolabeled tracers, such as PSMA ligands conjugated with radionuclides, to enhance real-time intraoperative detection of LNMs [13]. AR, on the other hand, provides a transformative tool for intraoperative navigation by overlaying three-dimensional (3D) preoperative imaging data, such as mpMRI or computed tomography (CT), onto the surgical field. This fusion of virtual and real-world visualization provides surgeons with a dynamic map, allowing for optimized NSS while minimizing the risk of PSMs [14].

This review aimed to synthesize current evidence on advanced technologies in robot-assisted PCa surgery, with a particular focus on the applicability of sentinel node biopsy (SNB), PSMA-RGS, and other types of fluorescence guidance for the intraoperative detection of LNMs, as well as the use of AR for avoiding PSMs during NSS, highlighting their pivotal role in driving the field toward a more precise, effective, and patient-centered surgical approach.

## 2. Review

A narrative review of the literature was conducted using the Web of Science, MEDLINE, the Cochrane Library’s CENTRAL, and EMBASE databases. We searched these databases from inception to January 2025. Additional sources included cross-referencing relevant cited references within identified articles. The search terms used were as follows: “(robot-assisted radical prostatectomy OR RARP) OR (prostate cancer OR PCa) AND ((PSMA radioguided surgery OR PSMA-RGS) OR (sentinel node biopsy OR SNB) OR (fluorescence-guided surgery OR fluorescence PSMA tracers) OR (augmented reality AND prostate cancer))”. The most relevant articles were identified with the aim of providing a comprehensive summary on the role of robot-assisted, imaging-guided surgery in PCa patients.

## 3. Sentinel Node Biopsy for Primary Staging

SNB represents one of the first attempts to use intraoperative guidance to identify the sentinel lymph node, which represents the first lymph node to receive drainage directly from a tumor [15]. SNB employs a range of tracers such as indocyanine green (ICG), radioactive tracers, or a hybrid fluorescent–radioactive ICG tracer. These tracers are typically administered into the peripheral zone of the prostate to aid surgical navigation, providing real-time guidance through either radioactivity or fluorescence [16]. Mazzone et al. [17] assessed the added value of SNB to ePLND for detecting LNMs. The authors relied on a cohort of 1168 patients who underwent ePLND at the time of RP. Among these, 351 men received ePLND combined with SNB using a hybrid fluorescent–radioactive tracer, while 161 patients underwent ePLND with SNB using ICG alone. The incidence of pN1 patients was 19% for ePLND alone vs. 28% for ePLND with SNB and the hybrid tracer vs. 36% for ePLND with SNB and ICG alone. In multivariable logistic regression analyses, use of SNB with the hybrid tracer was an independent predictor for identification of positive nodes at final pathology (Odds Ratio [OR]: 1.61; *p* = 0.002). Lannes et al. [18] provided further insight into the diagnostic accuracy of SNB through a prospective, single-arm, multicenter study including 162 PCa cN0M0 patients. SNB was performed utilizing a gamma probe following preoperative intraprostatic injection of ^99m^technetium-nanocolloid. Sentinel lymph nodes were successfully identified in 142 patients (88%), and LNMs were detected in 22 patients (14%). Among these, 17 cases (77%) were confined to the sentinel lymph nodes only. On a per-patient basis, SNB exhibited a sensitivity of 95.4%, a specificity of 100%, a positive predictive value (PPV) of 100%, and a negative predictive value (NPV) of 99%.

Overall, SNB represents a feasible technique for nodal staging during RARP with ePLND. However, it may fail to detect micro-metastases and skip metastases, leading to underestimation of disease burden. Skip metastases occur when PCa cells bypass the first expected lymph node that receives drainage from the tumor and spread directly to more distant lymphatic stations. This phenomenon challenges the assumption of SNB that the sentinel node reliably represents the first site of nodal involvement. Moreover, variability in lymphatic drainage patterns of the prostate could affect the accuracy of SNB, increasing rates of false-negative findings. Given these limitations, SNB should be interpreted with caution for nodal staging in PCa patients and still represents an experimental procedure.

## 4. PSMA-RGS: Rationale and Application

Approximately 20% of PCa patients undergoing RARP with ePLND are diagnosed with LNI upon final pathological examination [7]. In this context, risk stratification tools and predictive models, incorporating clinical, biopsy, and preoperative imaging parameters, showed strong calibration and discrimination in identifying men at higher risk of LNI. Despite this, over 50% of patients deemed candidates for ePLND are ultimately found to have no LNMs on final pathology [19]. This potential overtreatment raises significant concerns due to the extended operative time and the increased risk of complications associated with ePLND [20]. By enabling the precise identification of LNMs, PSMA-RGS has the potential to reduce unnecessary ePLNDs [21,22,23]. Moreover, the use of PSMA PET allowed for the early identification of metastases, which are typically located in the lymph nodes, in patients experiencing BCR after primary treatment [24]. The availability of an intraoperative guidance using PSMA-RGS can assist physicians in maximizing the chances of removing all metastatic sites during salvage lymph node dissection (sLND) [25,26,27]. The development and clinical application of PSMA-RGS represents an innovative and promising advancement in robot-assisted PCa surgery, specifically designed to enable the precise location of LNMs [13], thereby enhancing local disease control and improving oncological outcomes [16,28,29,30]. Differently from SNB, PSMA-RGS is a procedure that employs the use gamma-ray detectors to identify tissues with a high avidity for radiolabeled tracers, with the aim of providing immediate feedback to the surgeon on the presence of tumoral cells [16,30]. Radioactive labelling of PSMA ligands with gamma-emitting radionuclides, such as ^111^Indium-PSMA imaging and therapy ([^111^In]In-PSMA-I&T) or ^99m^Technetium-PSMA imaging and surgery ([^99m^Tc]Tc-PSMA-I&S), was established during the last few years [31,32]. [^99m^Tc]Tc-PSMA-I&S is the most commonly employed radioligand in clinical practice due to the fact that [^111^In]In-PSMA-I&T is limited due to its higher cost, inferior pharmacokinetic properties, increased radiation exposure, and limited availability [25,31,32]. Conversely, [^99m^Tc]Tc-PSMA-I&S represents a valuable option based on easy access to ^99m^Tc from ^99Mo/99m^Tc generators as routine equipment in nuclear medicine departments at a relatively low cost. Moreover, it has a strong in vivo stability and a relatively slow whole-body clearance due to high plasma protein binding, which promotes efficient tracer uptake in PCa lesions over time and leads to steadily increasing target-to-background (TtB) ratios up to 21 hours after injection [25,31]. [^99m^Tc]Tc-PSMA-I&S is usually administrated approximately 20 hours before surgery, and a single-photon emission CT/CT (SPECT/CT) imaging is subsequently performed to document positive tracer uptake within the lesions and to serve as a quality control. Historically, PSMA-RGS was first introduced in the context of open sLND [33]. However, the recent development of a drop-in gamma probe capable of detecting signals emitted from PSMA-avid tissues following the preoperative intravenous administration of radioligands has made PSMA-RGS feasible during robot-assisted surgery [27,34,35]. The drop-in gamma probe is typically introduced through a 15 mm assistant port positioned above the right iliac crest, and it is autonomously manipulated by the console surgeon using robotic prograsp forceps. The probe’s control unit provides both acoustic and numerical feedback in response to radioligand activity, facilitating precise identification of LNMs. A positive finding is usually defined as a TtB ratio at least twice that of the background reference, which is represented by the homolateral fatty tissue in each patient [25]. Additionally, ex vivo gamma measurements can also be performed to confirm the removal of the radioactive lesion or to prompt further searching in case of a missing signal [22,25]. Generally, the intraoperative measurement with the drop-in gamma probe is performed at the level of pelvic lymph node stations, aimed at guiding the removal of LNMs during PSMA-RGS. This technique first demonstrated its utility in identifying LNMs during sLND in BCR patients with positive PSMA PET after primary treatment, and it has also recently been described as identifying positive nodes in patients undergoing RARP.

### 4.1. PSMA-RGS for Recurrence Setting

sLND represents a potential treatment option for PCa patients experiencing BCR with suspicious LNMs at PSMA PET [36]. Bravi et al. [37] reported the long-term outcomes of sLNDs, showing limited cancer control, with a 10-year cancer-specific survival rate of 66%. However, these results may have been influenced by inadequate treatment planning due to the limitations of CIM. Unfortunately, a considerable proportion of patients still demonstrate a persistently elevated prostate specific antigen (PSA) level following sLND [37], which may be due to an incomplete resection of metastatic lesions [37,38]. In this context, two main challenges for the successful surgical treatment of these patients exist. First, correct assessment of the metastatic spread is crucial, and the introduction of PSMA PET has led to significant improvements in this regard. Second, as LNMs can atypically be located outside the standard template of an ePLND and/or are morphologically unremarkable at preoperative PSMA PET, reliable intraoperative identification and removal of LNMs are still challenging [25]. PSMA-RGS has been introduced in the setting of sLND to improve the intraoperative localization of LNMs. This approach was first described by Maurer et al. [33], who demonstrated the feasibility of PSMA-RGS. The same group also [25] reported the accuracy and short-term oncological outcomes of PSMA-RGS for sLND in 31 patients with recurrent PCa. Of the 132 specimens removed, 58 (43.9%) had LNMs at final pathology. According to PSMA-RGS, 120 specimens were suspicious for LNMs. No false-positive findings were reported, but 12 specimens were false-negatives. This resulted in sensitivity, specificity, PPV, and NPV of 84%, 100%, 100%, and 89%, respectively, with an accuracy of 93%. A PSA reduction to below 0.2 ng/mL was observed in 20 patients. Thirteen patients remained BCR-free after a median follow-up of 13.8 months (range: 4.6–18.3), and 20 patients remained BCR-free after a median follow-up of 12.2 (range: 5.5–18.3) months. Moreover, de Barros et al. [27] reported the results of the first prospective feasibility study using a drop-in gamma probe, assessing the performance characteristics of PSMA-RGS in 20 patients with up to 3 pelvic recurrences identified on PSMA PET following primary treatment (75% RP, 25% radiotherapy [RT]) undergoing sLND. No complications related to the drop-in gamma probe were observed. A total of 21 PSMA-avid lesions were pursued in 19 patients. The drop-in gamma probe was able to identify 19 out of 21 (90%) PCa lesions detected on PSMA PET. A total of 75 sites were excised, with 22 (29%) LNMs at final pathology. PSMA-RGS demonstrated a sensitivity, specificity, PPV, and NPV of 86%, 100%, 100%, and 95%, respectively. False-negative LNMs were all < 3 mm, consistent with the detection limits of the gamma probe previously described [21,22]. Additionally, Knipper et al. [39] recently described the largest cohort of patients undergoing PSMA-RGS sLND, evaluating the oncological outcome of 553 patients with one or more positive nodes at preoperative PSMA PET. Overall, 522 patients (94%) had metastatic tissue removed during the procedure. Multivariable analyses identified PSA levels of 0.1–0.2 ng/mL (hazard ratio [HR]: 1.9, 95% confidence interval [CI] 1.1–3.1) and PSA ≥ 0.2 ng/mL (HR 3.2, 95% CI: 2.2–4.6, *p* < 0.001) as independent predictors of subsequent treatment needs after PSMA-RGS. After 2 years of follow-up, the treatment-free survival (TFS) rates were 81%, 56%, and 43% (*p* < 0.001) for patients with PSA levels < 0.1, 0.1 – < 0.2, and > 0.2 ng/mL, respectively. To explore the concordance between suspicious LNMs at preoperative PSMA PET and positive finding during PSMA-RGS, Berrens et al. [40] also analyzed the correlation between the standardized uptake value maximum (SUVmax) of positive lymph nodes at preoperative PSMA PET and their intraoperative count rate during PSMA-RGS. They relied on 29 BCR patients after previous curative-intent therapy and a maximum of 3 positive pelvic lymph nodes at preoperative PSMA PET. The median count rates were 134 (range: 81–220) in vivo and 109 (range: 72–219) ex vivo. A significant correlation was observed between the intensity of the count rates and SUVmax (*ρS* = 0.728 and 0.763, respectively; *p* = 0.001). Subgroup analysis based on median SUVmax showed no correlation in the group with a SUVmax < 6 either with the in vivo count rate (*ρS* = 0.382; *p* = 0.221) or the TtB ratio (*ρS* = 0.245; *p* = 0.442). In contrast, the group with a SUVmax ≥ 6 showed statistically significant positive correlations (*ρS* = 0.774 [*p* < 0.001] and *ρS* = 0.647 [*p* < 0.007]). These results indicated that SUVmax could be used to optimize patient selection for PSMA-RGS sLND. Regarding this point, Falkenbach et al. [41] assessed the value of EAU BCR risk groups and PSA kinetics on PSMA-RGS outcomes. They evaluated 374 patients undergoing sLND with the use of PSMA-RGS for oligometastatic recurrence PCa after RP. The authors found that BCR risk groups were not associated with BCR-free survival (BCR-FS) (HR:1.61, CI: 0.70–3.71, *p* = 0.3) or TFS (HR:1.07, 95% CI: 0.46–2.47, *p* = 0.9). A total of 47 out of 76 (62%) patients with PSA-doubling time (DT) ≤ 6 months and 50/84 (60%) with PSA-DT > 6 months achieved complete biochemical response (cBR) (*p* = 0.4). Moreover, PSA-DT was not associated with cBR (OR: 0.99, 95% CI: 0.95–1.03, *p* = 0.5), BCR-FS (HR: 1.00, 95% CI: 0.97–1.03, *p* = 0.9), or TFS (HR: 1.02, 95% CI: 0.99–1.04, *p* = 0.2). Moreover, Falkenbach et al. [42] explored the feasibility of repeating PSMA-RGS sLND in patients who had previously undergone sLND with the support of PSMA-RGS or sLND alone. They identified 37 patients undergoing PSMA-RGS after prior sLND (*n* = 21) or prior PSMA-RGS (*n* = 16). At multivariable analysis, age (HR: 1.09, 95% CI 1.01–1.17) and preoperative PSA (HR: 1.23, 95% CI 1.01–1.50) were associated with shorter BCR-FS. Overall, one year after the second salvage surgery, 89% of the patients did not require additional treatment. Therefore, repeating PSMA-RGS could be safe and provide BCR-FS and TFS for selected cases. Patients having low preoperative PSA seemed to benefit most from repeating PSMA-RGS, irrespective of prior sLND or PSMA-RGS. Preliminary data are also present on the role of PSMA-RGS in patients experiencing local recurrence after primary treatment for PCa. To the best of our knowledge, only one study [43] assessed the outcomes of patients with local recurrence after RP who underwent salvage PSMA-RGS. The study included 40 patients with retrovesical or seminal vesicle bed recurrences identified on PSMA PET. Of these, 33 patients (82.5%) had previously undergone RT (12 patients [36.4%] received adjuvant RT, and 21 patients [63.6%] underwent salvage RT [sRT]). All resected specimens were positive for PCa. Complications were observed exclusively in 7 previously irradiated patients (17.5%), with 3 patients experiencing Clavien–Dindo grade III complications. A cBR was achieved in 78% of patients. The one-year BCR-FS and TFS were 62% and 88%, respectively, with a median BCR-FS of 24 months.

Overall, PSMA-RGS emerges as a promising therapeutic strategy for PCa patients experiencing recurrence after primary treatment (Table 1). By leveraging the precision of PSMA-targeted imaging, this technique enables the intraoperative identification and resection of LNMs during sLND. While early clinical outcomes suggest its feasibility and potential efficacy, long-term follow-up studies are needed to fully evaluate its impact on oncological control.

### 4.2. Initial Experience of PSMA-RGS for Primary Staging

PSMA-RGS was first described for nodal staging by Gondoputro et al. [21], who assessed that this technique is a safe and feasible approach for PCa patients undergoing RARP with ePLND. The study relied on 12 high-risk PCa patients, evaluating intraoperative drop-in gamma probe signals during PSMA-RGS compared with regional pathological results. In 7 patients, preoperative PSMA PET scans identified pelvic lymph node involvement across 11 distinct nodal sites. PSMA-RGS identified 32 positive stations (16 confirmed as PCa at final pathology) and 41 negative stations (36 without PCa), yielding in vivo sensitivity, specificity, PPV, and NPV of 76%, 69%, 50%, and 88%, respectively. Shortly after the first report was published, Gandaglia et al. [22] released the pre-planned interim analysis of the first 12 patients enrolled in a prospective study evaluating the feasibility and performance characteristics of PSMA-RGS for nodal stating during RARP with ePLND. Preoperative PSMA PET identified suspicious nodal uptake in 2 patients (17%). At a per-region analysis, PSMA-RGS achieved a sensitivity of 50%, specificity of 99%, a PPV of 80%, and an NPV of 96%. At per-patient analysis, the probe demonstrated a sensitivity, specificity, PPV, and NPV of 67%, 100%, 100%, and 90%, respectively. Additionally, Yilmaz et al. [44] conducted an additional evaluation of the accuracy of PSMA-RGS in 15 PCa patients who underwent RARP with ePLND. The study reported that PSMA-RGS achieved 100% sensitivity, specificity, NPV, and PPV on a per-node basis. Moreover, Schilham et al. [45] recently reported the safety and feasibility of PSMA-RGS in PCa patients who had one or more suspicious LNMs identified on preoperative PSMA PET. In 20 patients evaluated, 43 of 49 (88%) suspicious LNMs at PSMA PET were successfully removed. PSMA-RGS facilitated the perioperative identification and resection of 29 of 49 (59%) lesions, of which 28 (97%) were confirmed as LNMs at final pathology examination. An additional 14 of 49 (29%) resected lymph nodes were not detected by PSMA-RGS, two of which contained LNMs no larger than 3 mm in size. This resulted in a sensitivity, specificity, PPV, and NPV of PSMA-RGS (combining in vivo and ex vivo) on a per-lesion analysis of 66.7%, 99.8%, 96.8%, and 97.0%, respectively. Although all previously cited studies identified PSMA-RGS as a technically feasible surgical approach, the limited spatial resolution of the probe appeared to impact in vivo accuracy, as nodal metastases < 3 mm were missed in the Gondoputro et al. [21], Gandaglia et al. [22], and Schilham et al. [45] studies. This limitation may impact the performance of PSMA-RGS, especially due to the TtB ratio cut-offs used to define positive findings. Notably, both Gandaglia et al. [22] and Schilham et al. [45] defined intraoperative positivity as a TtB ratio of at least twice the background reference, whereas Gondoputro et al. [21] used a TtB ratio ≥ 1.5. In this context, Quarta et al. [23] investigated the performance and clinical implications of different TtB ratio thresholds (≥ 2 vs. ≥ 3 vs. ≥ 4) in a prospective study comparing intraoperative findings with preoperative PSMA PET. The authors found that preoperative PSMA PET combined with a TtB ratio ≥ 3 improved accuracy and reduced false-negatives compared with a TtB ratio ≥ 2. However, a TtB ratio ≥ 3 resulted in a significant increase in false-positive findings. Therefore, the authors recommended a TtB ratio ≥ 2 to improve the sensitivity of PSMA-RGS and reduce the risk of false-negatives. The available evidence suggests that PSMA-RGS is a safe and feasible approach in patients undergoing RARP with ePLND (Table 2). However, further studies are needed to better define its role and potential benefits in clinical practice. Preliminary data suggest that PSMA-RGS has the potential to enhance intraoperative nodal staging by improving the detection of LNMs. This capability may contribute to more accurate pathological staging, which is essential for tailoring adjuvant therapies and prognostic stratification. However, several questions remain regarding the long-term oncological benefits of PSMA-RGS. Specifically, it is unclear whether this technique might significantly impact long-term outcomes, such as BCR-FS, metastasis-free survival (MFS), or overall survival (OS), by enabling more complete removal of LNMs during RARP. Furthermore, the optimal selection criteria for identifying patients who would benefit the most from PSMA-RGS remain to be determined.

## 5. Fluorescence PSMA-Based Tracers: OLT78 & IS-002

Fluorescence-guided surgery using new tumor-specific tracers is a promising technique to identify PCa cells that exploits real-time surgical guidance [46,47]. Recently, two different near-infrared, fluorescently labeled, PSMA-targeting peptides have been proposed for intraoperative real-time visualization of PSMs and LNMs: OLT78 and IS-002 [48,49].

Here, a phase II trial [50] evaluated the safety, optimal dose, dosing interval, sensitivity, specificity, false-negative rate, and false-positive rate of OTL78 for fluorescence-guided surgery in PCa patients undergoing RARP with ePLND. OLT78 consists of a high-affinity PSMA-targeting ligand, namely 2-[3-(1,3-dicarboxypropyl)-ureido] pentanedioic acid (DUPA), conjugated to the near-infrared dye S0456 using a 14-atom-long polyethylene glycol–dipeptide linker [48]. The authors reported data from 18 patients undergoing RARP with ePLND using OTL78, which was administered using a single intravenous infusion. The outcomes evaluated were safety, pharmacokinetics, and diagnostic utility of the tracer across varying doses and intervals. A single intravenous dose of 0.03 mg/kg OTL78 administered 24 hours before surgery provided the highest signal-to-background ratio and the best tumor visualization both in vivo and ex vivo. During surgery, OTL78 allowed tumor visualization with adequate contrast to adjacent healthy tissue, facilitating the assessment of tumor resection margins. No drug-related serious adverse events were reported, with only one patient experiencing serious postoperative complications (Clavien-Dindo III) (infected lymphocele, urosepsis, and hemorrhage), which were considered as unrelated to the imaging agent. Regarding efficacy, OTL78 identified tumor-positive regions with high sensitivity both in vivo and ex vivo specimens, with the detection rate being higher in patients with higher PSMA expression, larger tumor volumes, and ISUP grade group ≥ 3. Additionally, OTL78 allowed in vivo visualization of LNMs. False-positive lymph nodes were observed only when patients received OTL78 on the day of surgery and not in the 24 hours dosing interval. Of note, immunohistochemistry showed no PSMA expression in false-positive lymph nodes, suggesting non-specific fluorescence staining occurred relatively shortly after administration. Indeed, no false-positive lymph node clusters were identified after the implementation of the 24 hours dose interval, probably due to increased wash-out of OTL78 from the lymphatic system. In this context, IS-002 represents a novel near-infrared fluorescent imaging agent composed of a urea-based PSMA-binding peptide and a near-infrared poly-methine cyanine dye. Nguyen et al. [49] assessed the role of IS-002 in detecting PSMs and LNMs in a phase 1 trial, including 24 high-risk PCa patients undergoing RARP with ePLND. No severe adverse events were reported, with the most frequent adverse event reported being temporary urine discoloration due to tracer’s renal clearance. Regarding the tracer’s performance characteristics, IS-002 exhibited a high NPV for locoregional disease (100%) and LNMs (97%). Similarly to what was observed by Stibbe et al. [50], false-positive findings were associated with tracer doses and timing of administration, with a higher risk of false-positive findings associated with higher tracer doses, suggesting an optimal dose of 25 μg/kg for the clear differentiation of cancerous tissues. In conclusion, this study suggests that IS-002 shows a promising ability to identify occult PSMs and micro-metastases, potentially reducing PSMs rates and improving LND staging accuracy.

Taken together, IS-002 and OTL78 reflect the remarkable progress in intraoperative imaging. Both agents may improve the visualization of cancer beyond what standard imaging modalities can achieve. IS-002 is notable for its versatility in highlighting residual disease in the prostate bed, while OTL78 might detect small LNMs. Moreover, they demonstrate excellent safety profiles, ensuring that their adoption into clinical practice would not pose additional risks to patients. Nonetheless, IS-002 requires a slightly higher dose than OTL78 to achieve its effects, but both emphasize the importance of minimizing background fluorescence for accurate imaging.

## 6. Augmented Reality

Intraoperative identification of the tumor margins during RARP might help surgeons to achieve complete resections, avoiding PSMs and allowing at the same time maximized functional outcomes through NSS. In this context, de Roode et al. described the use of diffuse reflectance spectroscopy (DRS), which measures tissue composition according to its optical properties, for real-time identification of PSMs during RARP [51]. This study aimed to evaluate DRS’s ability to differentiate PCa from benign tissue in RARP specimens. DRS measurements were taken ex vivo from 59 prostate specimens. A machine learning algorithm was developed using DRS spectral features to classify cancerous and healthy tissue. The data were divided into training (70%) and testing (30%) sets, with ten iterations to test the algorithm’s performance. A total of 542 DRS measurements were collected, 53% from cancerous tissue and 47% from healthy tissue. The model achieved an average sensitivity of 89%, specificity of 82%, accuracy of 85%, and an area under the curve (AUC) of 0.91, supporting a potential role of DRS in avoiding PSMs during RARP.

Moreover, there has recently been increasing interest in the application of 3D virtual reconstructions derived from two-dimensional (2D) cross-sectional preoperative imaging (e.g., mpMRI, CT) [52,53]. This approach enables surgeons to achieve a more comprehensive and detailed understanding of the surgical anatomy tailored to each individual case. To create these models, the first step is the production of high-quality 3D virtual tools that precisely reproduce the patients’ real anatomical details. Once the 3D virtual models are obtained, they can be applied in three different settings: cognitive procedures [54], printing and application to cognitive procedures [53], and AR procedures [14]. AR refers to systems where users directly view their environment while a constructed device superimposes additional information or images to create a blended view of the real environment with the overlaid display. In surgery, this involves the superimposition of preoperative or intraoperative images onto the operative field. This allows surgeons to utilize information obtained from preoperative imaging such as CT scan and mpMRI while performing robot-assisted surgery. The superimposed images could provide important anatomic landmarks for the identification of ideal resection planes that would otherwise be more difficult to identify [55]. The use of AR has been described in the setting of RARP for avoiding PSMs during NSS [56]. Porpiglia et al. [14] published the promising results of an innovative AR platform that allows surgeons to overlap intraoperatively, in real-time hyper accuracy, three-dimensional (HA3D) models specifically tailored to each case, relying on the preoperative prostate mpMRI images [57]. This system has proven to be accurate in the identification of ECE, both in static and dynamic phases of the intervention. The authors compared the accuracy of HA3D model in identifying the ECE location during the NSS, compared to 2D-based cognitive procedures. They relied on 40 PCa patients who underwent 3D-AR RARP or, in case of unavailability of this technology, 2D-cognitive RARP (20 vs. 20). A metallic clip was placed at the level of suspected ECE, based on images given by the 3D-AR or mpMRI report. For the 3D-AR group, at microscopic assessment, the presence of tumor cells was confirmed in the suspicious area in 95.4% of the cases, with ECE correctly identified in 100.0% of the cases. Of note, compared to the 2D MRI cognitive group, 3D-AR was superior for ECE detection during the NSS (100% vs. 47.0%; *p* < 0.05). These results suggest that elastic 3D virtual models correctly simulate prostate deformation during RARP, with lesion location correctly identified even during dynamic phases, leading to a potential reduction of PSMs rates, maximizing functional outcomes. However, the main limitation associated with HA3D models is represented by the need of the entire overlap process to be manually driven by an assistant placed near the surgical console. To overcome this limitation, Checcucci et al. [58] described the development of an automatic AR system, guided by artificial intelligence (AI), that dynamically superimposes images without the need of an assistant for the overlapping process. The authors evaluated the accuracy of this new 3D artificial intelligence guided AR system in the identification of residual tumor at the level of the preserved neurovascular bundles (NVBs) at the end of NSS, with selective AR-guided excisional biopsy performed in case of suspicion of residual disease. 3D-AR guided biopsies were negative in all pT2 patients, while presence of cancer was identified in 14 cases among pT3 patients (14/16; 87.5%). PSM rates were 0% and 7.1% for pT2 and pT3 patients (<3 mm, Gleason Score 3), respectively. In conclusion, the 3D-AR system allowed surgeons to identify the presence of tumor at the level of NVB in 87.5% of pT3 patients and to perform a 3D-guided tailored NSS even in locally advanced diseases. In this context, Bianchi et al. [59] developed a novel technique of intraoperative frozen section (IFS) targeted to the index lesion by using 3D-AR models in patients undergoing NSS during RARP. The study group consisted of 20 consecutive PCa patients undergoing NSS with 3D-AR guided IFS, with a control group of 20 patients matched after 1:1 propensity score matching. The AR-3D superimposed model was utilized to identify the location of the index lesion during RARP and to guide the critical surgical dissection steps, including the apex, bladder neck and NSS. Consequently, a sample from the periprostatic tissue was obtained from the area where the AR-3D model projected the index lesion and sent for IFS analysis. Overall, PSM rates were comparable between the two groups; PSMs at the level of the index lesion were significantly lower in patients referred to 3D guided IFS to the index lesion (5%) than those in the control group (20%; *p* = 0.01). Another study [56] assessed the role of 3D models in patients undergoing RARP, compared to a no-3D control group, evaluating differences in PSM rates. A total of 160 patients were enrolled in the 3D group, while 640 were included in the control group. Patients treated with 3D-guided surgery had lower PSM rates, compared to the control group (25 vs. 35.1%, *p* = 0.01). At multivariable logistic regression models, use of 3D technology (*p* = 0.005) achieved independent predictor status for lower PSM risk after surgery.

The integration of AR with 3D virtual models during RARP has shown promising results for avoiding PSMs. Moreover, 3D-AR virtual models have the potential to enhance surgical precision during RARP, making patients with advanced disease or ECE the ideal candidates to gain the most benefit from these new surgical tools.

## 7. Conclusions

Imaging-guided surgery will have a role in enhancing the outcomes of PCa patients undergoing robot-assisted surgery soon. The integration of real-time intraoperative guidance into the surgical workflow represents a pivotal advancement in the precision medicine scenario. Different imaging-guided surgical approaches have been recently proposed, with the aim of intraoperatively assisting surgeons. Among these advancements, PSMA-RGS might offer an accurate LNMs detection, improving LND quality both in primary and recurrence settings by providing the precise localization of PSMA-avid tissue. This capability could reduce the rate of incomplete LNDs, thus improving oncological outcomes. Similarly, SNB could also improve nodal staging during RARP. Emerging technologies such as near-infrared fluorescent PSMA (OLT78 and IS-002) also represent promising intraoperative guidance. These agents have demonstrated excellent safety profiles and a potential role in enhancing intraoperative identification of LNMs. Additionally, AR plays a crucial role in optimizing NSS by providing real-time, high-resolution visualization of critical anatomical structures, allowing the avoidance of PSMs and potentially improving both oncological and functional outcomes. The integration of these cutting-edge technologies reflects a paradigm shift toward a more personalized and precise surgical approach, redefining standards of care in robot-assisted PCa surgery. However, several limitations must be acknowledged. Firstly, regarding PSMA-RGS, the lack of standardized protocols for imaging agents such as tracer selection and dosing intervals may introduce variability and limit its reproducibility. Regarding SNB, its use is subject to biological variability in lymphatic drainage and further constrained by the absence of standardized intraoperative mapping. Fluorescent PSMA-based tracers are still under early clinical investigation. Regarding AR, current systems rely on preoperative imaging that must be manually registered to the intraoperative anatomy, which can be invalidated by patient positioning and intraoperative tissue manipulation.

Looking ahead, further investigations should focus on refining tracer pharmacokinetics and standardized imaging protocols to increase intraoperative LNMs detection, as well as on integrating AI into AR platforms to improve real-time anatomical assessment and decision-making support. Lastly, prospective multicenter studies with long-term oncological and functional endpoints are needed to validate these technologies, optimize patient selection, and facilitate their integration into standardized surgical workflows.

## Figures and Tables

**Table 1:** Milestone studies regarding the applicability of PSMA-RGS in the recurrence setting of PCa.

Author	Year of Publication, Study Design	Patients Included	Tracer Used	Objectives	Results
Maurer et al. [25]	2019, retrospective	31 patients with recurrent PCa at PSMA PET after RP	[^99m^Tc]Tc-PSMA-I&S	to describe feasibility and short-term outcomes of PSMA-RGS for removal of recurrent PCa lesions	per-lesion analysis: - sensitivity: 83.6% - specificity: 100% - PPV: 100% - NPV: 89.2% 2-years BCR-FS: 43% 2-years TFS: 65%
De Barros et al. [27]	2022, prospective	20 patients with up to 3 pelvic PCa lesions at PSMA PET after RP or RT	[^99m^Tc]Tc-PSMA-I&S	to evaluate whether the drop-in gamma probe facilitates the use of PSMA-RGS in recurrent PCa patients	per-lesion analysis: - sensitivity: 86% - specificity: 100% - PPV: 100% - NPV: 95%
Knipper et al. [39]	2024, retrospective	553 oligorecurrent PCa patients with BCR and positive PSMA PET after RP	[^99m^Tc]Tc-PSMA-I&S, [^111^In]In-PSMA-I&T	to evaluate whether a PSA < 0.1 ng/mL is predictive of TFS following salvage PSMA-RGS	2-years TFS: 81.1% vs. 56.1% vs. 43.1% (*p* < 0.001) in patients with PSA < 0.1 vs. 0.1–<0.2 vs. >0.2 ng/mL, respectively

PCa = prostate cancer; PSMA = prostate-specific membrane antigen; PSA = prostate specific antigen; PET = positron emission tomography; RP = radical prostatectomy; RT = radiotherapy RGS = radioguided surgery; sLND = salvage lymph node dissection; BCR = biochemical recurrence; BCR-FS = biochemical recurrence-free survival; TFS = treatment-free survival; PPV = positive predictive value; NPV = negative predictive value; [^111^In]In-PSMA-I&T = ^111^Indium-PSMA imaging and therapy; [^99m^Tc]Tc-PSMA-I&S = ^99m^Technetium-PSMA imaging and surgery.

**Table 2:** Milestone studies regarding the applicability of PSMA-RGS for nodal staging of PCa.

Author	Year of Publication, Study Design	Patients Included	Tracer Used	Objectives	Results
Gondoputro et al. [21]	2022, prospective	12 HR-PCa patients with a LNI risk > 10%	[^99m^Tc]Tc-PSMA-I&S	to evaluate the safety and feasibility of PSMA-RGS to guide the intraoperative detection of LNMs during RARP with ePLND	per-lesion analysis: - sensitivity: 76% - specificity: 69% - PPV: 50% - NPV: 88%
Gandaglia et al. [22]	2022, prospective	12 IR- or HR- cN0cM0 PCa patients at CIM with a LNI risk > 5%	[^99m^Tc]Tc-PSMA-I&S	to report the planned interim analyses of a phase 2 prospective study aimed at describing PSMA-RGS during RARP with ePLND	per-lesion analysis: - sensitivity: 50% - specificity: 99% - PPV: 80% - NPV: 96%
Schilham et al. [45]	2024, prospective	20 PCa patients with positive preoperative PSMA PET	[^111^In]In-PSMA-I&T	to evaluate the safety and feasibility of PSMA-RGS during RARP with ePLND	per-lesion analysis: - sensitivity: 66.7% - specificity: 99.8% - PPV: 96.8% - NPV: 97%

PCa = prostate cancer; IR = intermediate risk; HR = high risk; LNI = lymph node invasion; LNM = lymph node metastasis; RARP = robot-assisted radical prostatectomy; ePLND = extended pelvic lymph node dissection; CIM = conventional imaging modality; PSMA = prostate-specific membrane antigen; PET = positron emission tomography; RGS = radioguided surgery; PPV = positive predictive value; NPV = negative predictive value; [^111^In]In-PSMA-I&T = ^111^Indium-PSMA imaging and therapy; [^99m^Tc]Tc-PSMA-I&S = ^99m^Technetium-PSMA imaging and surgery.

## Abbreviations

The following abbreviations are used in this manuscript:

AI	Artificial Intelligence
AR	Augmented Reality
AUC	Area Under Curve
BCR	Biochemical Recurrence
BCR-FS	Biochemical Recurrence-Free Survival
cBR	Complete Biochemical Response
CI	Confidence Interval
CIM	Conventional Imaging Modality
CT	Computed Tomography
DRS	Diffuse Reflectance Spectroscopy
ECE	Extra-Capsular Extension
ePLND	Extended Pelvic Lymph Node Dissection
HA3D	Hyper Accuracy Three-Dimensional
HR	Hazard Ratio
ICG	Indocyanine Green
IFS	Intraoperative Frozen Section
[^111^In]In-PSMA-I&T	^111^Indium-PSMA Imaging and Therapy
LNM	Lymph Node Metastasis
LNI	Lymph Node Invasion
MFS	Metastasis-Free Survival
mpMRI	Multiparametric Magnetic Resonance Imaging
NPV	Negative Predictive Value
NSS	Nerve Sparing Surgery
NVB	Neuro Vascular Bundle
OR	Odds Ratio
OS	Overall Survival
PCa	Prostate Cancer
PET	Positron Emission Tomography
PPV	Positive Predictive Value
PSA	Prostate Specific Antigen
PSM	Positive Surgical Margin
PSMA	Prostate-Specific Membrane Antigen
RARP	Robot-Assisted Radical Prostatectomy
RGS	Radioguided Surgery
RP	Radical Prostatectomy
RT	Radiotherapy
sLND	Salvage Lymph Node Dissection
SNB	Sentinel Node Biopsy
SUVmax	Standardized Uptake Value Maximum
SVI	Seminal Vesicle Invasion
[^99m^Tc]Tc-PSMA-I&S	^99m^Technetium-PSMA Imaging and Surgery
TFS	Treatment-Free Survival
TtB	Target-to-Background
2D	Two-Dimensional
3D	Three-Dimensional
